# Horizontal gene transfer in silkworm, *Bombyx mori*

**DOI:** 10.1186/1471-2164-12-248

**Published:** 2011-05-19

**Authors:** Bo Zhu, Miao-Miao Lou, Guan-Lin Xie, Guo-Qing Zhang, Xue-Ping Zhou, Bin Li, Gu-Lei Jin

**Affiliations:** 1 State Key Laboratory of Rice Biology and Key Laboratory of Molecular Biology of Crop Pathogens and Insects, Ministry of Agriculture, Institute of Biotechnology, Zhejiang University, Hangzhou 310029, China; 2 Institute of Bioinformatics, Zhejiang University, Hangzhou 310029, China

**Keywords:** *Bombyx mori*, horizontal gene transfer, phylogeny, functional analysis

## Abstract

**Background:**

The domesticated silkworm, *Bombyx mori*, is the model insect for the order Lepidoptera, has economically important values, and has gained some representative behavioral characteristics compared to its wild ancestor. The genome of *B. mori *has been fully sequenced while function analysis of *BmChi-h *and *BmSuc1 *genes revealed that horizontal gene transfer (HGT) maybe bestow a clear selective advantage to *B. mori*. However, the role of HGT in the evolutionary history of *B. mori *is largely unexplored. In this study, we compare the whole genome of *B. mori *with those of 382 prokaryotic and eukaryotic species to investigate the potential HGTs.

**Results:**

Ten candidate HGT events were defined in *B. mori *by comprehensive sequence analysis using Maximum Likelihood and Bayesian method combining with EST checking. Phylogenetic analysis of the candidate HGT genes suggested that one HGT was plant-to- *B. mori *transfer while nine were bacteria-to- *B. mori *transfer. Furthermore, functional analysis based on expression, coexpression and related literature searching revealed that several HGT candidate genes have added important characters, such as resistance to pathogen, to *B. mori*.

**Conclusions:**

Results from this study clearly demonstrated that HGTs play an important role in the evolution of *B. mori *although the number of HGT events in *B. mori *is in general smaller than those of microbes and other insects. In particular, interdomain HGTs in *B. mori *may give rise to functional, persistent, and possibly evolutionarily significant new genes.

## Background

The silkworm, *Bombyx mori*, was domesticated over 5,000 years ago and is well-known for its industrial importance in sericulture [1]. *B. mori *has become a model organism for studying other Lepidoptera insects that cause serious agricultural damage and is also an important model for scientific discovery in the areas of microbiology, physiology, and genetics. However, compared to its wild ancestor, *B. mori *has gained some representative behavioral characteristics such as tolerance to human proximity and handling, as well as extensive crowding and lost other traits such as flight, predators, and diseases avoidance [2].

Horizontal gene transfer (HGT) has been not only regarded as a driving force in the innovation and evolution of genomes in prokaryotes, but also plays an important role in eukaryotes [3,4]. In some specific eukaryotes (such as rotifers), HGT also serves as a important evolutionary impetus [5,6]. However, in most cases, the transferred genes in insect genomes are absence of function [7]. In contrast, Daimon *et al. *(2003, 2008) found that HGT maybe bestow a clear selective advantage to *B. mori *based on function analysis of *BmChi-h *and *BmSuc1 *genes, which have been cloned from *B. mori *[8,9] The first gene contributed the potential fungi resistance to *B. mori *while the latter gene serves as a sugar-digesting enzyme which can degrade the alkaloidal sugar mimic glycosidase inhibitors that are toxic to *B. mori*. These results indicated that the HGTs in *B. mori *are different from those of other insects, which may play important function in the evolution of *B. mori*.

Some methods such as GC content and codon usage have been applied in the detection of gene transfers, but these methods have been demonstrated to be unreliable without phylogenetic analysis [10,11]. Indeed, the detection of gene transfers is best achieved by generation of a strongly supported phylogenetic tree which contradicts the known species phylogeny [4,10]. Luckily, the genome of *B. mori *has been recently fully sequenced [8]. This provides a strong basis for the overall understanding of the existence and functions of HGTs in *B. mori *based on phylogenetic analysis.

In this study, the HGT events between *B. mori *and other unrelated species were investigated by sequence comparison of the entire predicted proteomes from *B. mori *with the genome sequences of 87 eukaryotic and 295 prokaryotic (Additional file 1), which represent a wide taxonomic diversity, and generated individual phylogenetic trees by different models combining with other evidences for every gene that showed a higher similarity to Arthropoda than those of other genomes. This process identified 10 putative gene transfers. Analysis of expression and coexpression revealed that these HGTs could enhance the disease resistance ability, nutrient and energy metabolism and toxin degradation. These studies give us a first glance to understand the HGTs in the evolution of *B. mori *by whole genome analysis.

## Methods

### Genome

The genome of *Bombyx mori *has a size of 428.7 Mb and is composed of 28 chromosomes [12]. Gene candidates for HGT in *B. mori *were identified by screening the 14,623 coding genes downloaded from SilkDB [13].

### Local database generating

In order to construct the phylogenetic trees for each sequence identified with the target taxon/similarity profile automatically and preciously, a local database (300 cores Processor BladeSystem in Zhejiang University, IBM-Biocomputing Laboratory) containing predicted protein sequences (2,385,947 coding sequences in total) from 382 species representing a wide diversity of eukaryotes and prokaryote taxa including seven insects, seven plants, 43 fungi, 30 other eukaryotes and 295 prokaryotes (Additional file 1).

### Search procedure

**Figure 1:**
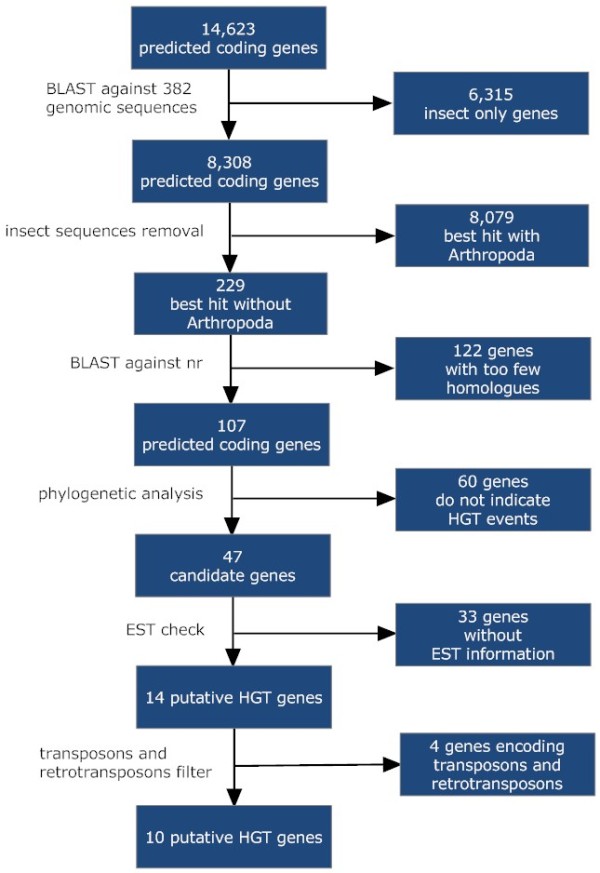
**Flowchart of the methodology used to search for HGT genes in *Bombyx mori *and of the results of each step**.

Each candidate sequence in *B. mori *was compared against sequences in the local database using BLASTp [14] and the highest similarity sequences from each species were extracted for further analysis (*E*-value cutoff 10^-20^). We used the local database first to exclude insect unique genes and the genes that, when compared against all the genomes showed a higher BLASTp similarity score to Arthropoda than to any other species (Figure 1). All sequences that had a high sequence similarity to the transposon were also excluded from further analysis as we aimed to study the effect of HGT on the putative functional genes (Figure 1). After detecting the putative transferred genes, expression sequence tags (EST) information was used to remove out the unexpressed genes. The detailed procedure was showed in Figure 1.

### Phylogenetic analysis

The remaining candidate HGT genes were selected to search against GenBank non-redundant protein database (nr). Search strategy was the same as described above. These sequences were aligned using ClustalW [15], the conserved region of each alignment was trimmed using Gblocks [16] with a stringent settings described previously [17]. Maximum Likelihood (ML) phylogenies were constructed by Phyml [18] using a JTT + Γ + I substitution model (Γ + I parameters were estimated by Phyml). The proportion of invariant sites was estimated from the data. For Bayesian phylogenies generated by MrBayes [19], two independent Metropolis-coupled Monte Carlo Markov Chain (MCMC) runs, each with one cold and three heated chains (heat parameter = default), were analyzed for one million generations after a burn-in of 25,000 samples and allowed mixed models of amino acid substitution. For Maximum Likelihood phylogenies, 1,000 bootstraps were performed to gain the branch support values. To be considered as an indicative of a potential HGT event, the ML branch support about *B. mori *should ≥ 80% while Bayesian posterior probability should ≥ 85%.

To test the support for contentious topology, we performed nonparametric branch support tests based on a Shimodaira-Hasegawa-like procedure using Tree-Puzzle [20]. In addition, we used this software to test the statistical significance (5%) of specific topology over a collapsed version of the same branching relationship.

### Functional analysis

To test whether the transferred genes were associated with the domestication of *B. mori*, single nucleotide polymorphism (SNP) genelist [2] produced by resequencing 40 *B. mori *genomes was used to search if the candidate genes are on the list. Meanwhile, expression and coexpression analysis about the transferred genes were also conducted. A Pearson correlation coefficient was calculated based on microarray gene expression profiling, which were downloaded from SilkDB [13] to investigate the function of transferred genes analyzed in this paper. Background correction and data normalization was done by RMA algorithm in Bioconductor [21] and the poor annotated probe-sets were removed. Measurements for unique gene was calculated from means of the probes belong to same gene. The gene coexpressed with one of either gene in ten putative transferred genes with the absolute correlation |r| > 0.5 was further selected to analyze the function. For each transferred gene, we obtained the top 300 genes in each list sorted by absolute correlation value r referred to as coexpressed genes in descending order. The distribution of coexpressed genes with each transferred gene was mapped onto pathways. A score reflecting the extent of coexpression in each gene was assigned to each pathway by using following formula: , where *G *is the number of genes in a pathway present in the top 300 of all same transferred genes. *R*_*i *_is the rank of the ith gene of the specified pathway in a list, *r*_*i *_is the absolute correlation value r.

## Results and Discussion

### The process of HGT candidate search

Two horizontally transferred genes have been previously described in *B. mori *[8,9]. In this paper, an exhaustive analysis was used to investigate any other HGT candidates. This study used a pipeline with several filters to search for *B. mori *predicted coding genes that are candidates obtained by HGT as summarized in Figure 1. In the first filter, the 14,623 predicted coding genes of *B. mori *were compared with 382 genome sequences, which excluded 6,315 genes as possible HGT candidates since they have homologs only in insect genomes. A total of 8,308 remaining proteins with their correspondent homologs were clustered using OrthoMCL [22] to group together potential orthologs (default parameters). In the second filter, the insect sequences in these orthologs were removed except *B. mori*. Every predicted *B. mori *protein sequence that showed a higher BLAST similarity score to a gene from any other species rather than Arthropoda was retained for subsequent analysis. This filter screen resulted in 229 clusters which were considered to be candidate *B. mori *HGTs. In the third filter, the 229 candidate HGT genes in *B. mori *were submitted to a BLASTp search against nr database, producing two categories of results. The first category consists of 122 few-hit genes, which were meaningless to construct the phylogenetic trees (Fewer than 5 genes in one phylogeny). The second category comprised the remaining 107 predicted genes with enough homologs. These genes were considered reliable HGT candidates. In the forth filter, phylogenetic trees were generated to test the possible HGTs. The criteria used in this procedure as indicative of HGT is the candidate gene should group with sequences from a non-related species with the gene on a well supported clade. The 107 HGT candidates produced 60 phylogenies that did not support the criteria (Figure 1). The remained 47 candidate HGT genes were validated by Bayesian and Maximum Likelihood phylogenetic methods (both methods inferred the same topology). In all these data sets, the SH test also showed the HGT topology at the 5% significance level [23]. As in this study, only functional genes were of our interest. So, in the last filter, *B. mori *EST database was used to remove the unfunctional genes, whereas transposons and retrotransposons were also excluded from further analysis. In order to reduce the errors, Chinese and Japanese *B. mori *EST databases were both applied [12,24]. The EST procedure reduced 33 genes while the latter procedure reduced 4 genes. This resulted in 10 putative HGT data sets, which were selected for further analysis. The general information about the 10 genes was presented in Table 1 and the phylogenetic trees about these genes were shown in Supplementary Figures. In additional, we also performed a blast against the *B. mori *genome itself to verify if those candidates could be duplicates. However, all of the 10 genes have no homologes out of themselves.

**Table 1:** HGT candidates in the *Bombyx mori *genome.

Gene name	Gene ID	Scaffold	Position	Top Hit organism *	Accession number	*E*-value/Similarity/Coverage	SH test $
NAD-dependent epimerase/dehydratase	BGIBMGA010285	chr7	10699008-10699964	*Providencia rustigianii*	ZP_05974183.2	2e-103/75%/98%	1.000
Glycerophosphoryl diester phosphodiesterase	BGIBMGA007767	chr15	9903542-9906121	*Pseudomonas aeruginosa*	NP_249038.1	7e-135/80%/89%	1.000
chitinase	BGIBMGA008709	chr7	14117862-14119517	*Serratia proteamaculans*	YP_001476375.1	0/81%/99%	1.000
glucose-1-phosphatase/inositol phosphatase	BGIBMGA011204	chr23	19418818-19419798	*Edwardsiella tarda*	YP_003295806.1	2e-85/67%/99%	0.974
sucrose-6-phosphate hydrolase	BGIBMGA005696	nscaf2830	395557-397023	*Bacillus licheniformis*	YP_081250.1	2e-101/61%/88%	1.000
aromatic ring-opening dioxygenase LigB subunit	BGIBMGA003842	chr24	16178938-16179756	*Talaromyces stipitatus*	XP_002485623.1	4e-65/66%/90%	0.969
alginate lyase	BGIBMGA005615	chr17	3487011-3488021	*Bacillus halodurans*	NP_241604.1	3e-52/74%/100%	1.000
kynureninase	BGIBMGA007146	chr21	17770472-17771752	*Listeria grayi*	ZP_07053169.1	4e-159/77%/100%	0.989
N-methyltryptophan oxidase	BGIBMGA008215	chr18	14492832-14493941	*Serratia odorifera*	ZP_06189604.1	1e-120/71%/99%	1.000
gamma-glutamyltranspeptidase	BGIBMGA002521	chr9	16188873-16190453	*Serratia odorifera*	ZP_06637018.1	8e-176/75%/100%	0.999

* The insect sequences were removed from top hit organism

^$ ^5% confidence level was used in SH test

In this study, stringent filters were used to identify the real HGT events. Although this procedure could identify the really HGT event undoubtedly, it may neglect several potential HGT events. First, the HGT in the common ancestor of *Insecta *may be considered as vertical inheritance in this study if none of the other sampled taxons has homolog. As no homologs were detected outside insects, it might be assumed that this HGT was from an extinct linkages [25]. However, this assumption can only been proved by fossil evidence. Second, our filter could also remove genes transferred from other animal to *B. mori *as we only retained the genes showed a higher BLAST similarity score to a gene from any other species rather than Arthropoda (Material and Methods). However, fewer instances of potential HGTs between eukaryotes are known [4,26], and most of these HGTs were plastids-eukaryote transfer. Finally, we only retained the transferred genes that have putative functions as functional genes may play more important role in physical and biochemical reaction in *B. mori*.

Comparative phylogenetic analysis, which included models that account for site rate heterogeneity and, where appropriate, comparative topology tests were used in our study. We finally identified 10 genes that was the most consistent explanation to have been transferred from the genome of bacteria to the genome of *B. mori*. The number of transferred genes is nearly the same as recently *Acyrthosiphon pisum *sequencing paper [7]. The direction of transfer from prokaryote to *B. mori *is also highly suggested in our analysis. As in the phylogenetic trees, *B. mori *nested within a bacterial clade, and many bacterial species formed basel branches. Meanwhile, all of these 10 genes are intronless in the *B. mori*. Based on these results, the direction of transfer from prokaryote to *B. mori *is mainly confirmed. Recent findings suggested that substantial HGT between prokaryotic endosymbionts and their insect hosts were always happened [7,27-32]. However, in this study, none of the putative transferred gene was acquired from a previous commonly reported endosymbiont (*Wolbachia *or *Buchnera*).

### The HGT events in *B. mori *are rare but recent

It has long been described that many insects such as pea aphid, mosquitoes, and fruit flies have been influenced by HGT from *Wolbachia *[33]. Nikoh *et al. *have suggested that nearly 30% of a *Wolbachia *genome was found on the X-chromosome of the insect, probably as the result of a single HGT event [30]. In this study, we only get 10 HGTs in silkworm. Compared to previous reports, we may think that the HGT events are rare compared to other insects. The CDS of 10 putative transferred genes were used to search against wildsilkbase [34], which is a BLAST searchable catalogue of ESTs generated from three major wild silkworms, *Antheraea assama*, *Samia cynthia ricini *and *Antheraea mylitta*. We found that the transferred genes in *B. mori *were actually absent in three wild silkmoths. This phenomenon can be explained by two hypotheses. The first suggests that the aliened genome fragment was transferred to the *B. mori *chromosome after speciation of *Bombycoidea*, while the latter suggests an ancient HGT in the common ancestor with several losses. However, the "losses" hypothesis is highly unlikely for two reasons. It requires massive independent losses to explain the presence of these genes only in *B. mori*. Furthermore, this hypothesis has difficulty to explain the remarkable sequence identity between *B. mori *and its candidate donors (Table 1), while the general gene similarity between wild and domesticated silkworm is 58.6% based on the protein sequences of *Antheraea assama *deposited in Genbank. Taking into account, it appears most likely that the aliened genome fragment was transferred to the *B. mori *chromosome after speciation of *Bombycoidea*. The result suggested that these HGT events may be happened much recently. The recovery of only 10 potential HGT in *B. mori *suggests that HGT events happened in this species have played only a very minor role in its evolution (0.065% in the *B. mori *protein coding genes). Interestingly, none of the HGT events involved donor linkages from the common ancestor of *Insecta*. Instead, all of the HGTs involved transfer from bacteria or plant directly to the *Bombycidae *except chitinase, which has been demonstrated to be an ancient HGT event [9]. It is worth noting that half of these transfers originate from the *Enterobacteriaceae *species. Many *Enterobacteriaceae *species have been demonstrated to be as symbionts of insects [35]. It may facilitate to exchange the genetic material between symbionts and hosts.

### Putative functional assignment of 10 HGTs

The potential identities and biological functions of the 10 HGT candidates were investigated based on the similarity of their putatively encoded products to known proteins. We found that the 10 putative transferred genes were all enzymes, which suggested a strong functional trend among these HGTs in *B. mori*. Furthermore, hypergeometric distribution analysis of the candidate HGT genes indicated catalytic activity and hydrolase activity genes are significantly overrepresented (with p-values 4.6e^-05 ^and 6.4e^-06^, respectively). Compared with our study, none of the putative functional gene was identified in Aphid genome sequence analysis [7]. The acquisition of such genes by HGT may therefore have been selected by a competitive advantage in the recipient to degrade or take up substrates with greater efficiency.

**Figure 2:**
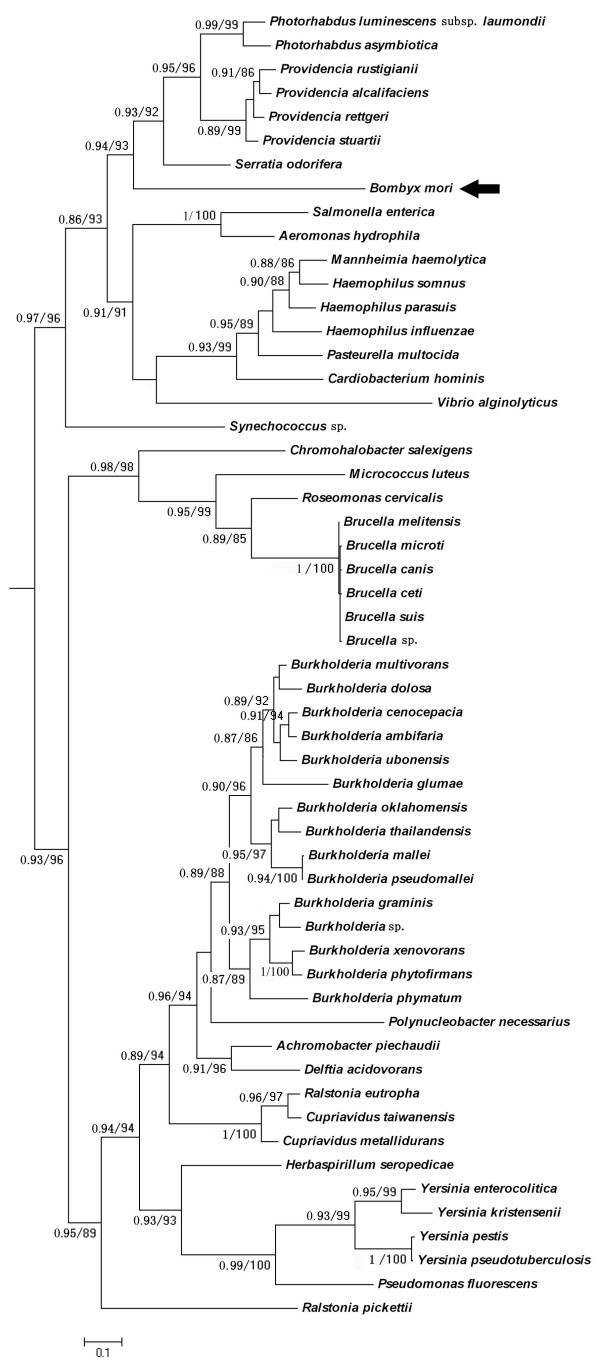
**A phylogeny of the putative NAD-dependent epimerase/dehydratase (BGIBMGA010285)**. The phylogenetic tree shown was calculated using the Maximum Likelihood (ML) program PhyML and Bayesian program Mr. bayes (detailed parameters were described in the main text of paper). Only the values in ML ≥ 80% and Bayesian posterior probability ≥85% were shown. For key nodes the actual support values are shown in the order ML bootstraps/Bayesian posterior probability. Bar, 0.1 substitution per site.

**Figure 3:**
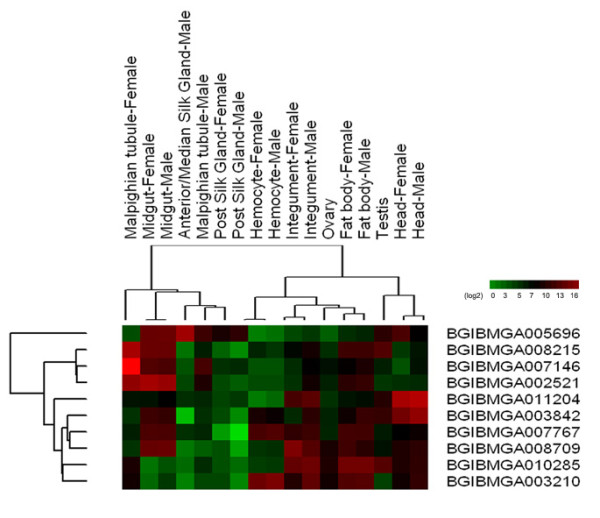
**Heatmap about tissue specifity expression analysis of transferred genes in *Bombyx mori***. Microarray expression data were retrieved from the SilkDB database and processed as described in Methods.

One HGT data set (Figure 2) transferred from bacteria to *B. mori *in this study is a enzyme encoding NAD-dependent epimerase/dehydratase (BGIBMGA010285, pfam01370, e-value 3.2e^-25^), which catalyses the last step in biosynthesis of GDP-d-rhamnose in bacteria [36]. This kind of gene has been demonstrated to be transferred in chromalveolates [37]. Expression analysis suggested that this gene was highly expressed in integument and fat body (Figure 3), whereas coexpression analysis revealed that this gene may be associated with biotin metabolism with high value support (Additional file 2). As insect store energy reserves mainly in fat body cells [38] and biotin metabolism is related to silk synthesis by nutrient affection [39]. Based on these results, we can infer that this gene may be associated with nutrient and energy metabolism in *B. mori*.

A putative glycerophosphoryl diester phosphodiesterase (BGIBMGA007767, pfam03009, e-value 1e^-52^) was encoded by an HGT candidate from the *Pseudomonas *species to *B. mori *(Additional file 3). This enzyme display broad specificity for glycerophosphodiesters, glycerophosphocholine, glycerophosphoethanolamine, glycerophosphoglycerol and bis (glycerophosphoglycerol), all of which are hydrolysed by this enzyme. This gene also has been reported to be involved in HGT between spider and bacteria [40]. Expression analysis suggested that this gene was not only highly expressed in integument and fat body but also in midgut and hemocyte. Coexpression analysis indicated that the function of this gene may be consistent with BGIBMGA010285.

Two genes, encoded chitinase and sucrose-6-phosphate hydrolase, are the only two genes which have been reported to be transferred from bacteria to *B. mori *previously [8,9] were also identified in this study. *B. mori *acquired the first gene from a bacterium or an ancestral baculovirus and the latter gene from a bacterium [8,9]. The results suggested that both genes bestow a clear selective advantage to *B. mori*. The discovery of both genes indicated that our strategy in searching candidate HGTs in *B. mori *is reliable.

Another candidate HGT was a gene transferred from *Edwardsiella tarda *(Additional file 4). Homology analysis suggested that this gene was a glucose-1-phosphatase/inositol phosphatase (BGIBMGA011204, pfam00328, e-value 1.4e^-17^). This enzyme has been reported to enhance the phytase activity in bacteria [41]. Expression pattern revealed that this gene is extremely highly expressed in head (Figure 2). Coexpression analysis suggested that the function of this gene may be most associated with Butirosin and neomycin biosynthesis (Additional file 2). As the gene in this pathway is involved in aminoglycoside antibiotics biosynthesis, which is effective against several types of bacteria [42]. The result indicated that this gene may be associated with the resistance of *B. mori *to bacterial pathogen.

The sixth HGT candidate (Additional file 5) showed sequence similarity to alginate lyase (BGIBMGA005615, pfam08240, e-value 2.1e^-08^), which can catalyze the degradation of alginate by a β-elimination mechanism [43]. In bacteria, this enzyme has been reported to destroy the cell detachment, which is the key process in biofilm formation [44]. Unfortunately, there is no corresponding probe to detect this gene in microarray data.

The seventh, eighth and ninth HGT are putative kynureninase (BGIBMGA007146, pfam00266, e-value 2.6e^-18^), N-methyltryptophan oxidase (BGIBMGA008215, pfam01266, e-value 4.2e^-54^) and gamma- glutamyltranspeptidase (pfam01019, e-value 4.3e^-125^), respectively. The seventh gene encoded by an HGT candidate from *Listeria grayi *to *B. mori *(Additional file 6). The mutation of this gene has been reported to be associated with abnormal body coloration in *B. mori *[45]. The eighth and ninth genes were both encoded by an HGT candidate from *Enterobacteriaceae *to *B. mori *(Additional file 7 and 8). In both genes, the eighth one has been reported to be involved in substrate specificity determination [46] while the ninth gene plays a key role in the gamma-glutamyl cycle, a pathway for the synthesis and degradation of glutathione and drug and xenobiotic detoxification [47]. All of the three genes are highly expressed in malpighian tubule (Figure 2), and the coexpression patterns are similar (Additional file 2). The results indicated that these genes may be associated with valine, leucine and isoleucine degradation pathway (Additional file 2). It has been reported that after *B. mori *was infected by its bacterial pathogen *Bacillus bombyseptieus*, the expression level of the genes which are associated with valine, leucine and isoleucine degradation pathway were more than three times the baseline levels in the malpighian tubule. Huang *et al*. (2009) speculated that these genes might be involved in the detoxification of malpighian tubules [48]. The results suggested that these genes may be associated with the resistance of *B. mori *to bacterial pathogen.

The tenth gene which encoded as aromatic ring-opening dioxygenase LigB subunit (BGIBMGA003842, pfam02900, e-value 1.2e^-51^) was transferred from a plant donor (Additional file 9). No tissue specific expression pattern was observed in this gene (Figure 2). However, coexpression analysis suggested that this gene may be most associated with Butirosin and neomycin biosynthesis (Additional file 2). It has been reported that Butirosin and neomycin belong to a family of clinically valuable 2-deoxystreptamine (2DOS)-containing aminoglycoside antibiotics [49]. The result indicated that this gene may be associated with the resistance of *B. mori *to bacterial pathogen.

In this study, the coexpressed genes involved in HGT are mapped onto biological pathway to investigate the potential function in *B. mori*. This scheme provides a comprehensive way to identify previously unknown functional patterns in sets of genes with known functions. It has been well known that the domesticated *B. mori *has gained or lost some representative behavioral characteristics compared to its wild ancestor. A lot of researches have focused on the search for 'domestication genes' in mammals by the polygenic nature of most behavioral traits [50]. Indeed, the linking of coat-color genes in mammals to brain biochemistry and behaviors has been found to be able to facilitate domestication and ease of handling [50]. In our study, SNP data searching indicated that all of the 10 genes have no direct relationship with human domestication although all of these genes are suggested to be transferred recently after the divergence from the wild silkworm. Therefore, we could suggest that HGT in *B. mori *maybe mainly depend on its nature requirement, but not on human activity. However, our study strongly suggested that these transferred genes increase the ability of *B. mori *to natural survival and adaption.

## Conclusion

In this study, we conclude that HGT is both rare and recent between *B. mori *and other unrelated species based on large-scale genome sequence data analysis using a strict and highly conservative set of ML and Bayesian methods combining with other analysis such as EST checking for inferring HGTs. Results from this study clearly indicated that HGT in *B. mori *has occurred and may have provided advantageous gene functions that could enhance the disease resistance ability, nutrient and energy metabolism and toxin degradation.

## Authors' contributions

BZ performed the analysis and wrote the manuscript, MML collected the information used in this study and wrote the manuscript, GLX, GQZ and XPZ were responsible for revising this manuscript, BL contributed to the work, GLJ conceived the work and wrote the manuscript. All the authors discussed the results, commented on the manuscript and approved the final manuscript.

## Supplementary Material

Additional file 1 **Genomes used for HGT identification pipeline**. Click here for file

Additional file 2 **The results about mapping coexpressed genes with each transferred gene onto pathways**. Click here for file

Additional file 3 **Phylogenies of other first reported putative transferred genes in *Bombyx mori *in this paper**. Click here for file

Additional file 4 **Phylogenies of other first reported putative transferred genes in *Bombyx mori *in this paper**. Click here for file

Additional file 5 **Phylogenies of other first reported putative transferred genes in *Bombyx mori *in this paper**. Click here for file

Additional file 6 **Phylogenies of other first reported putative transferred genes in *Bombyx mori *in this paper**. Click here for file

Additional file 7 **Phylogenies of other first reported putative transferred genes in *Bombyx mori *in this paper**. Click here for file

Additional file 8 **Phylogenies of other first reported putative transferred genes in *Bombyx mori *in this paper**. Click here for file

Additional file 9 **Phylogenies of other first reported putative transferred genes in *Bombyx mori *in this paper**. Click here for file

## References

[B1] Goldsmith MR Shimada T Abe H The genetics and genomics of the silkworm, *Bombyx mori* Annu Rev Entomol 2005 50 71 100 10.1146/annurev.ento.50.071803.130456 15355234

[B2] Xia QY Guo YR Zhang Z Li D Xuan ZL Li Z Dai FY Li YR Cheng DJ Li RQ Complete Resequencing of 40 Genomes Reveals Domestication Events and Genes in Silkworm (*Bombyx*) Science 2009 326 433 436 10.1126/science.1176620 19713493 PMC3951477

[B3] Boucher Y Douady CJ Papke RT Walsh DA Boudreau MER Nesbo CL Case RJ Doolittle WF Lateral gene transfer and the origins of prokaryotic groups Annu Rev Genet 2003 37 283 328 10.1146/annurev.genet.37.050503.084247 14616063

[B4] Keeling PJ Palmer JD Horizontal gene transfer in eukaryotic evolution Nat Rev Genet 2008 9 605 618 10.1038/nrg2386 18591983

[B5] Kaessmann H Origins, evolution, and phenotypic impact of new genes Genome research 2010 20 1313 1326 10.1101/gr.101386.109 20651121 PMC2945180

[B6] Gladyshev EA Meselson M Arkhipova IR Massive horizontal gene transfer in bdelloid rotifers Science 2008 320 1210 1213 10.1126/science.1156407 18511688

[B7] Nikoh N McCutcheon JP Kudo T Miyagishima S Moran NA Nakabachi A Bacterial Genes in the Aphid Genome: Absence of Functional Gene Transfer from *Buchnera *to Its Host PLoS Genet 2010 6 10.1371/journal.pgen.1000827 PMC2829048 20195500

[B8] Daimon T Taguchi T Meng Y Katsuma S Mita K Shimada T Beta-fructofuranosidase genes of the silkworm, *Bombyx mori *- Insights into enzymatic adaptation of *B. mori *to toxic alkaloids in mulberry latex J Biol Chem 2008 283 15271 15279 10.1074/jbc.M709350200 18397891 PMC3258877

[B9] Daimon T Hamada K Mita K Okano K Suzuki MG Kobayashi M Shimada T A *Bombyx mori *gene, BmChi-h, encodes a protein homologous to bacterial and baculovirus chitinases Insect Biochem Mol Biol 2003 33 749 759 10.1016/S0965-1748(03)00084-5 12878222

[B10] Richards TA Soanes DM Foster PG Leonard G Thomton CR Talbot NJ Phylogenomic Analysis Demonstrates a Pattern of Rare and Ancient Horizontal Gene Transfer between Plants and Fungi Plant Cell 2009 21 1897 1911 10.1105/tpc.109.065805 19584142 PMC2729602

[B11] Ragan MA On surrogate methods for detecting lateral gene transfer FEMS Microbiol Lett 2001 201 187 191 10.1111/j.1574-6968.2001.tb10755.x 11470360

[B12] Xia QY Zhou ZY Lu C Cheng DJ Dai FY Li B Zhao P Zha XF Cheng TC Chai CL A draft sequence for the genome of the domesticated silkworm (*Bombyx mori*) Science 2004 306 1937 1940 15591204 10.1126/science.1102210

[B13] Wang J Xia QY He XM Dai MT Ruan J Chen J Yu G Yuan HF Hu YF Li RQ SilkDB: a knowledgebase for silkworm biology and genomics Nucleic Acids Res 2005 33 D399 D402 15608225 10.1093/nar/gki116 PMC540070

[B14] Altschul SF Madden TL Schaffer AA Zhang JH Zhang Z Miller W Lipman DJ Gapped BLAST and PSI-BLAST: a new generation of protein database search programs Nucleic Acids Res 1997 25 3389 3402 10.1093/nar/25.17.3389 9254694 PMC146917

[B15] Thompson JD Higgins DG Gibson TJ ClustalW - improving the sensitivity of progressive multiple sequence alignment through sequence weighting, position-specific gap penalties and weight matrix choice Nucleic Acids Res 1994 22 4673 4680 10.1093/nar/22.22.4673 7984417 PMC308517

[B16] Castresana J Selection of conserved blocks from multiple alignments for their use in phylogenetic analysis Mol Biol Evol 2000 17 540 552 10742046 10.1093/oxfordjournals.molbev.a026334

[B17] Ciccarelli FD Doerks T von Mering C Creevey CJ Snel B Bork P Toward automatic reconstruction of a highly resolved tree of life Science 2006 311 1283 1287 10.1126/science.1123061 16513982

[B18] Guindon S Gascuel O A simple, fast, and accurate algorithm to estimate large phylogenies by maximum likelihood Syst Biol 2003 52 696 704 10.1080/10635150390235520 14530136

[B19] Ronquist F Huelsenbeck JP MrBayes 3: Bayesian phylogenetic inference under mixed models Bioinformatics 2003 19 1572 1574 10.1093/bioinformatics/btg180 12912839

[B20] Schmidt HA Strimmer K Vingron M von Haeseler A TREE-PUZZLE: maximum likelihood phylogenetic analysis using quartets and parallel computing Bioinformatics 2002 18 502 504 10.1093/bioinformatics/18.3.502 11934758

[B21] Gentleman RC Carey VJ Bates DM Bolstad B Dettling M Dudoit S Ellis B Gautier L Ge YC Gentry J Bioconductor: open software development for computational biology and bioinformatics Genome Biol 2004 5 10.1186/gb-2004-5-10-r80 PMC545600 15461798

[B22] Li L Stoeckert CJ Roos DS OrthoMCL: Identification of ortholog groups for eukaryotic genomes Genome Res 2003 13 2178 2189 10.1101/gr.1224503 12952885 PMC403725

[B23] Shimodaira H Hasegawa M CONSEL: for assessing the confidence of phylogenetic tree selection Bioinformatics 2001 17 1246 1247 10.1093/bioinformatics/17.12.1246 11751242

[B24] Mita K Morimyo M Okano K Koike Y Nohata J Kawasaki H Kadono-Okuda K Yamamoto K Suzuki MG Shimada T The construction of an EST database for *Bombyx mori *and its application Proc Natl Acad Sci USA 2003 100 14121 14126 10.1073/pnas.2234984100 14614147 PMC283556

[B25] Fournier GP Huang JL Gogarten JP Horizontal gene transfer from extinct and extant lineages: biological innovation and the coral of life Philos Trans R Soc Lond B Biol Sci 2009 364 2229 2239 10.1098/rstb.2009.0033 19571243 PMC2873001

[B26] Andersson JO Lateral gene transfer in eukaryotes Cell Mol Life Sci 2005 62 1182 1197 10.1007/s00018-005-4539-z 15761667 PMC11138376

[B27] Woolfit M Iturbe-Ormaetxe I McGraw EA O'Neill SL An Ancient Horizontal Gene Transfer between Mosquito and the Endosymbiotic Bacterium *Wolbachia pipientis* Mol Biol Evol 2009 26 367 374 10.1093/molbev/msn253 18988686

[B28] Kondo N Nikoh N Ijichi N Shimada M Fukatsu T Genome fragment of *Wolbachia *endosymbiont transferred to X chromosome of host insect Proc Natl Acad Sci USA 2002 99 14280 14285 10.1073/pnas.222228199 12386340 PMC137875

[B29] Hotopp JCD Clark ME Oliveira D Foster JM Fischer P Torres MC Giebel JD Kumar N Ishmael N Wang SL Widespread lateral gene transfer from intracellular bacteria to multicellular eukaryotes Science 2007 317 1753 1756 10.1126/science.1142490 17761848

[B30] Nikoh N Tanaka K Shibata F Kondo N Hizume M Shimada M Fukatsu T Wolbachia genome integrated in an insect chromosome: Evolution and fate of laterally transferred endosymbiont genes Genome Res 2008 18 272 280 10.1101/gr.7144908 18073380 PMC2203625

[B31] Klasson L Kambris Z Cook PE Walker T Sinkins SP Horizontal gene transfer between *Wolbachia *and the mosquito *Aedes aegypti* BMC Genomics 2009 10 10.1186/1471-2164-10-33 PMC2647948 19154594

[B32] Nikoh N Nakabachi A Aphids acquired symbiotic genes via lateral gene transfer BMC Biol 2009 7 10.1186/1741-7007-7-12 PMC2662799 19284544

[B33] Hotopp D Julie C Horizontal gene transfer between bacteria and animals Trends Genet 2011 27 157 163 10.1016/j.tig.2011.01.005 21334091 PMC3068243

[B34] Arunkumar KP Tomar A Daimon T Shimada T Nagaraju J WildSilkbase: An EST database of wild silkmoths BMC Genomics 2008 9 10.1186/1471-2164-9-338 PMC2483293 18637161

[B35] Moran NA Russell JA Koga R Fukatsu T Evolutionary relationships of three new species of *Enterobacteriaceae *living as symbionts of aphids and other insects Appl Environ Microbiol 2005 71 3302 3310 10.1128/AEM.71.6.3302-3310.2005 15933033 PMC1151865

[B36] Anderson I Sorokin A Kapatral V Reznik G Bhattacharya A Mikhailova N Burd H Joukov V Kaznadzey D Walunas T Comparative genome analysis of *Bacillus cereus *group genomes with *Bacillus subtilis* FEMS Microbiol Lett 2005 250 175 184 10.1016/j.femsle.2005.07.008 16099605

[B37] Nosenko T Bhattacharya D Horizontal gene transfer in chromalveolates BMC Evol Biol 2007 7 10.1186/1471-2148-7-173 PMC2064935 17894863

[B38] Arrese EL Soulages JL Insect Fat Body: Energy, Metabolism, and Regulation Annu Rev Entomol 2010 55 207 225 10.1146/annurev-ento-112408-085356 19725772 PMC3075550

[B39] Zhou ZH Yang HJ Chen M Lou CF Zhang YZ Chen KP Wang Y Yu ML Yu F Li JY Zhong BX Comparative Proteomic Analysis between the Domesticated Silkworm (*Bombyx mori*) Reared on Fresh Mulberry Leaves and on Artificial Diet J Proteome Res 2008 7 5103 5111 10.1021/pr800383r 18998723

[B40] Cordes MHJ Binford GJ Lateral gene transfer of a dermonecrotic toxin between spiders and bacteria Bioinformatics 2006 22 264 268 10.1093/bioinformatics/bti811 16332712

[B41] Herter T Berezina OV Zinin NV Velikodvorskaya GA Greiner R Borriss R Glucose-1-phosphatase (AgpE) from *Enterobacter cloacae *displays enhanced phytase activity Appl Microbiol Biotechnol 2006 70 60 64 10.1007/s00253-005-0024-8 16193276

[B42] Kudo F Eguchi T Biosynthetic genes for aminoglycoside antibiotics J Antibiot 2009 62 471 481 10.1038/ja.2009.76 19644520

[B43] Wong TY Preston LA Schiller NL Alginate lyase: Review of major sources and enzyme characteristics, structure-function analysis, biological roles, and applications Annu Rev Microbiol 2000 54 289 340 10.1146/annurev.micro.54.1.289 11018131

[B44] Boyd A Chakrabarty AM RRole of alginate lyase in cell detachment of *Pseudomonas aeruginosa* Appl Environ Microbiol 1994 60 2355 2359 8074516 10.1128/aem.60.7.2355-2359.1994 PMC201655

[B45] Meng Y Katsuma S Mita K Shimada T Abnormal red body coloration of the silkworm, *Bombyx mori*, is caused by a mutation in a novel kynureninase Genes Cells 2009 14 129 140 10.1111/j.1365-2443.2008.01257.x 19170761

[B46] Ilari A Bonamore A Franceschini S Fiorillo A Boffi A Colotti G The X-ray structure of N-methyltryptophan oxidase reveals the structural determinants of substrate specificity Proteins Struct Funct Bioinformat 2008 71 2065 2075 10.1002/prot.21898 18186483

[B47] Courtay C Oster T Michelet F Visvikis A Diederich M Wellman M Siest G Gamma-glutamyl-transferase - nucleotide-sequence of the human pancreatic cdna - evidence for a ubiquitous gamma-glutamyl-transferase polypeptide in human tissues Biochem Pharmacol 1992 43 2527 2533 10.1016/0006-2952(92)90140-E 1378736

[B48] Huang LL Cheng TC Xu PZ Cheng DJ Fang T Xia QY A Genome-Wide Survey for Host Response of Silkworm, *Bombyx mori *during Pathogen *Bacillus bombyseptieus *Infection PLoS One 2009 4 10.1371/journal.pone.0008098 PMC2780328 19956592

[B49] Kudo F Eguchi T Biosynthetic enzymes for the aminoglycosides butirosin and neomycin Methods Enzymol 2009 459 493 591 19362652 10.1016/S0076-6879(09)04620-5

[B50] Price EO Animal domestication and behavior 2002 Wallingford Oxon: Cab International

