# Supplementary figures and images for: Horizontal gene transfer in silkworm, Bombyx mori
Source: BMC Genomics. 2011 May 19;12:248. doi: 10.1186/1471-2164-12-248 (PMC3116507; doi:10.1186/1471-2164-12-248)

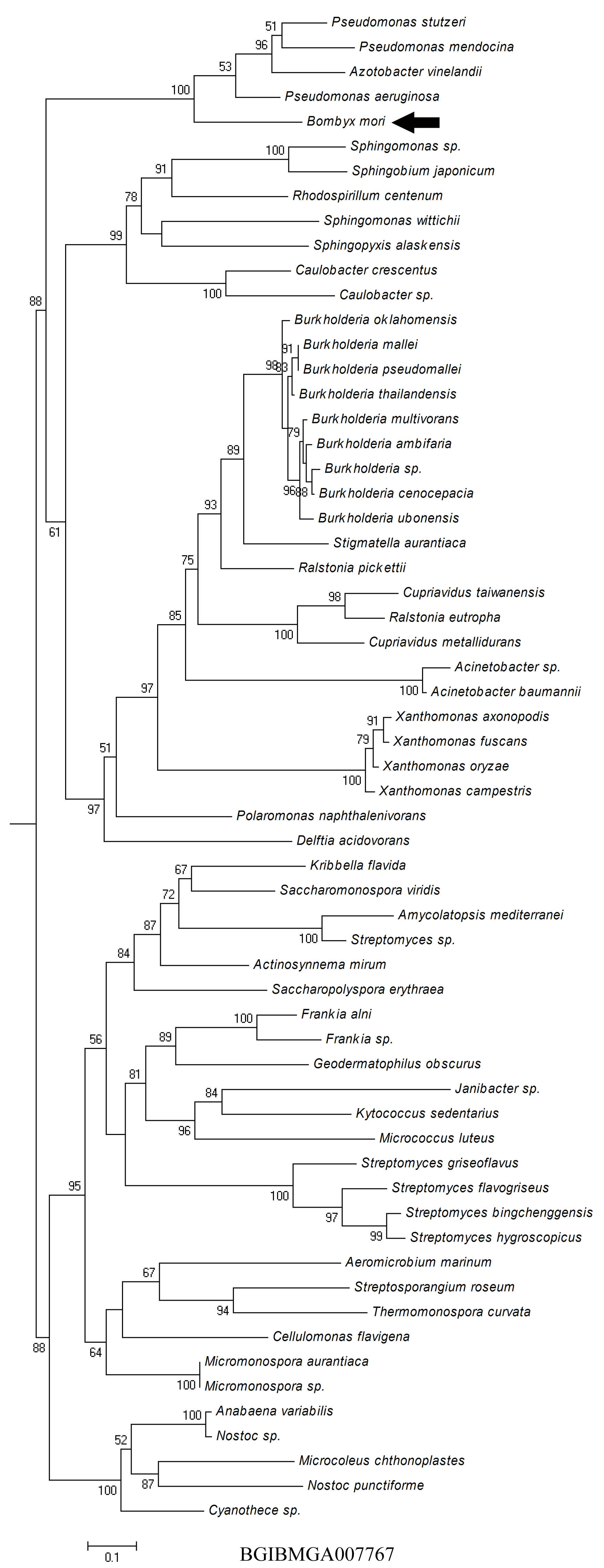

Supplement: Additional file 3 — Phylogenies of other first reported putative transferred genes in Bombyx mori in this paper. [file 1471-2164-12-248-S3.JPEG]

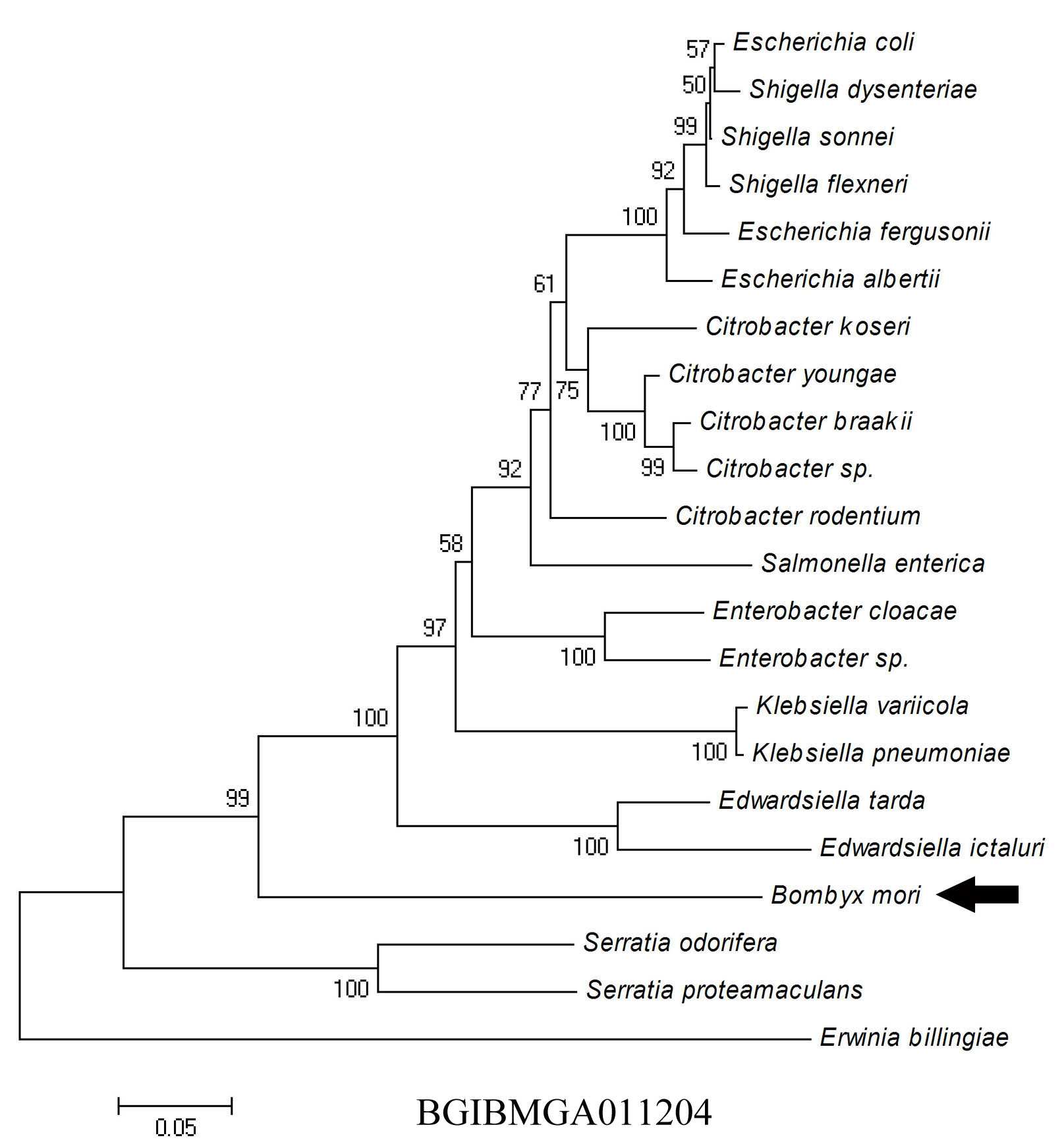

Supplement: Additional file 4 — Phylogenies of other first reported putative transferred genes in Bombyx mori in this paper. [file 1471-2164-12-248-S4.JPEG]

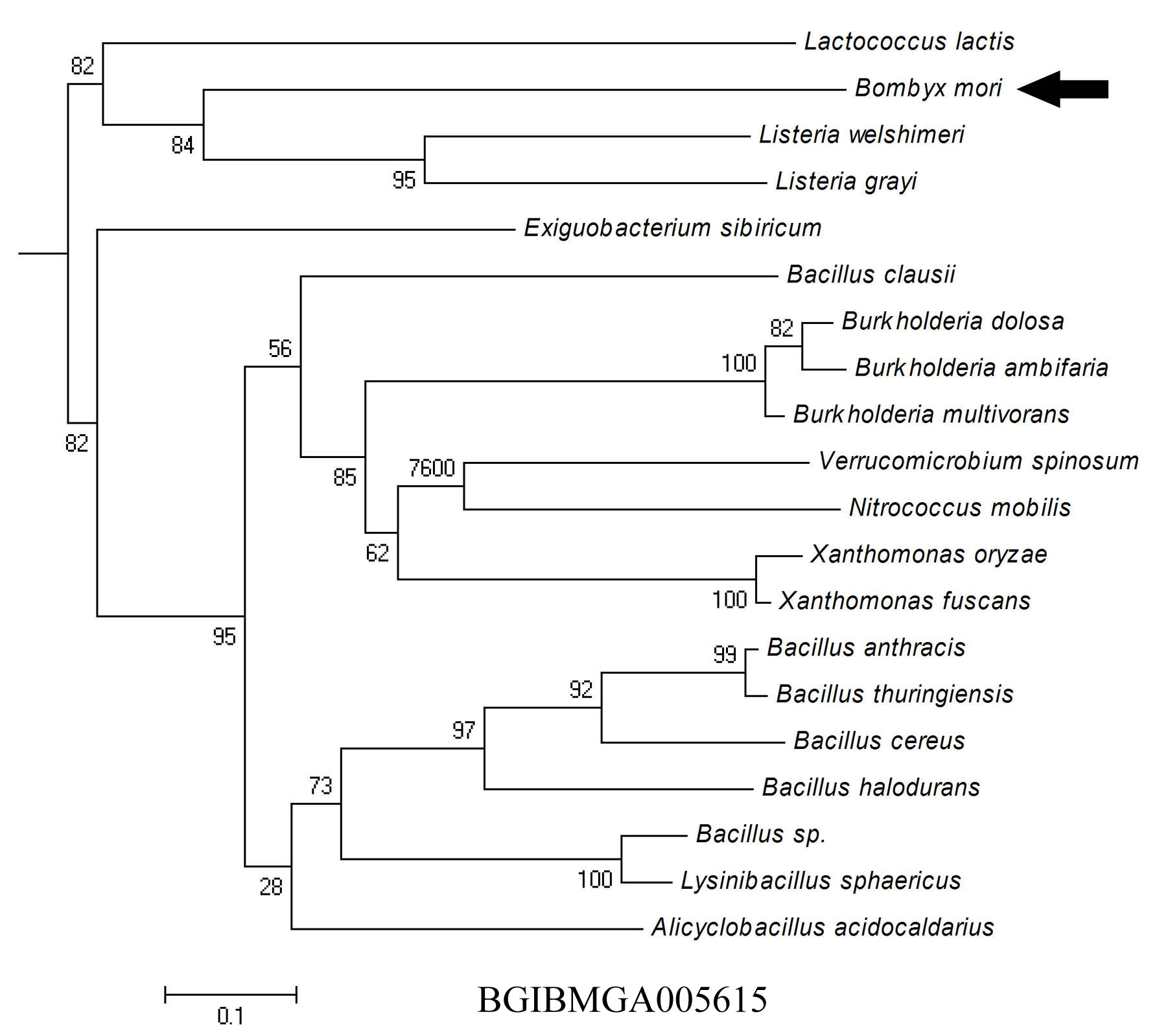

Supplement: Additional file 5 — Phylogenies of other first reported putative transferred genes in Bombyx mori in this paper. [file 1471-2164-12-248-S5.JPEG]

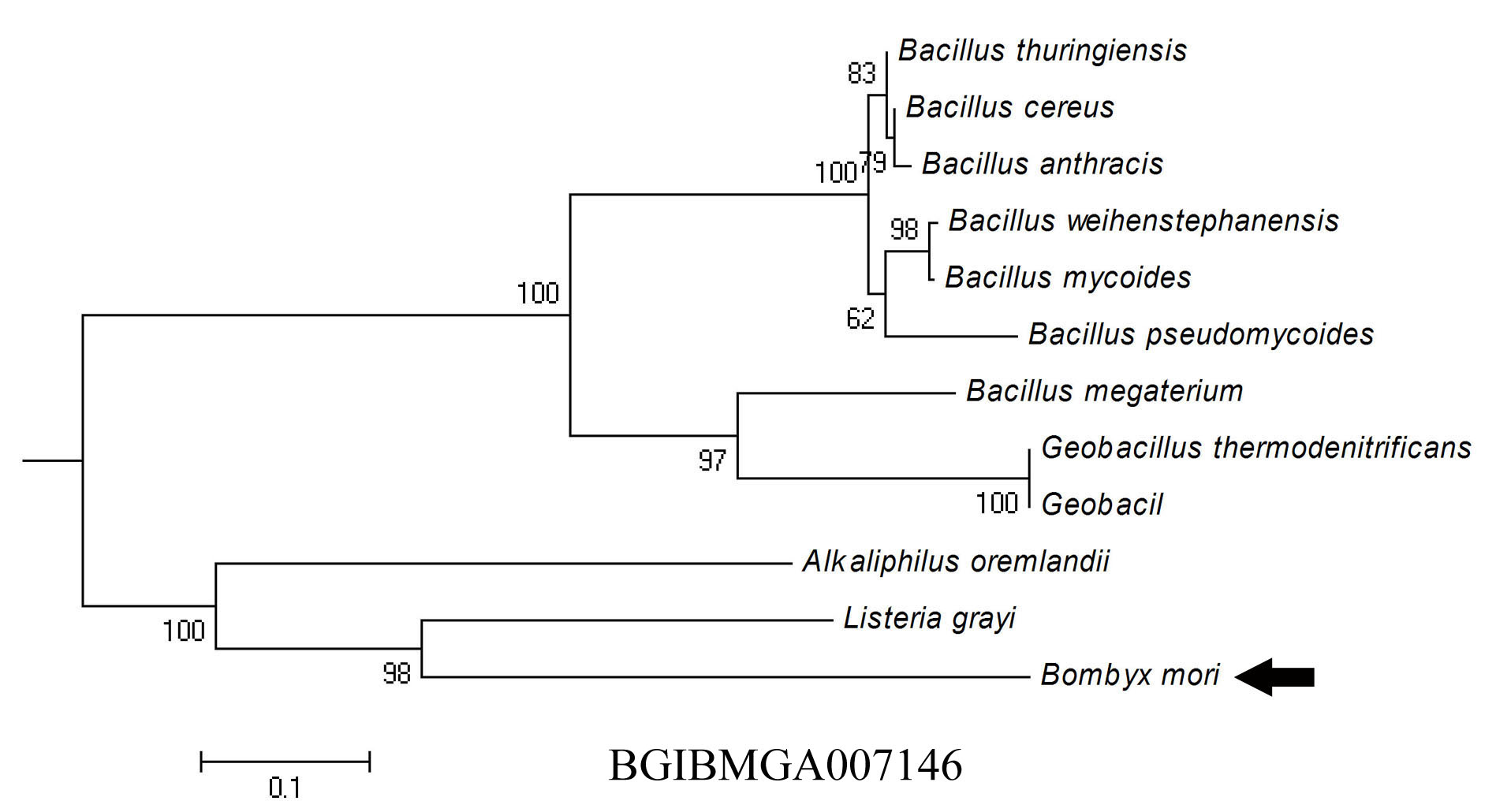

Supplement: Additional file 6 — Phylogenies of other first reported putative transferred genes in Bombyx mori in this paper. [file 1471-2164-12-248-S6.JPEG]

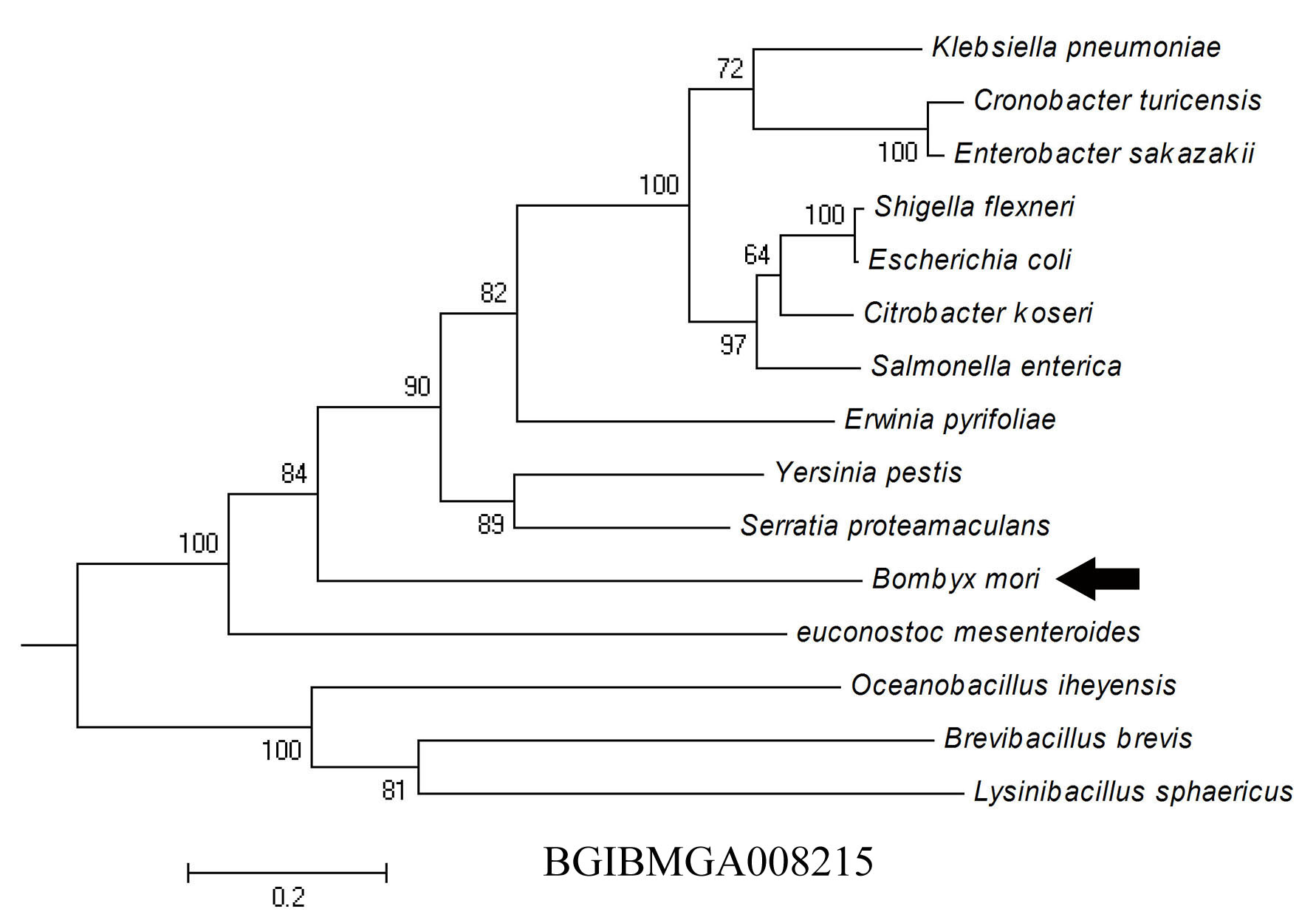

Supplement: Additional file 7 — Phylogenies of other first reported putative transferred genes in Bombyx mori in this paper. [file 1471-2164-12-248-S7.JPEG]

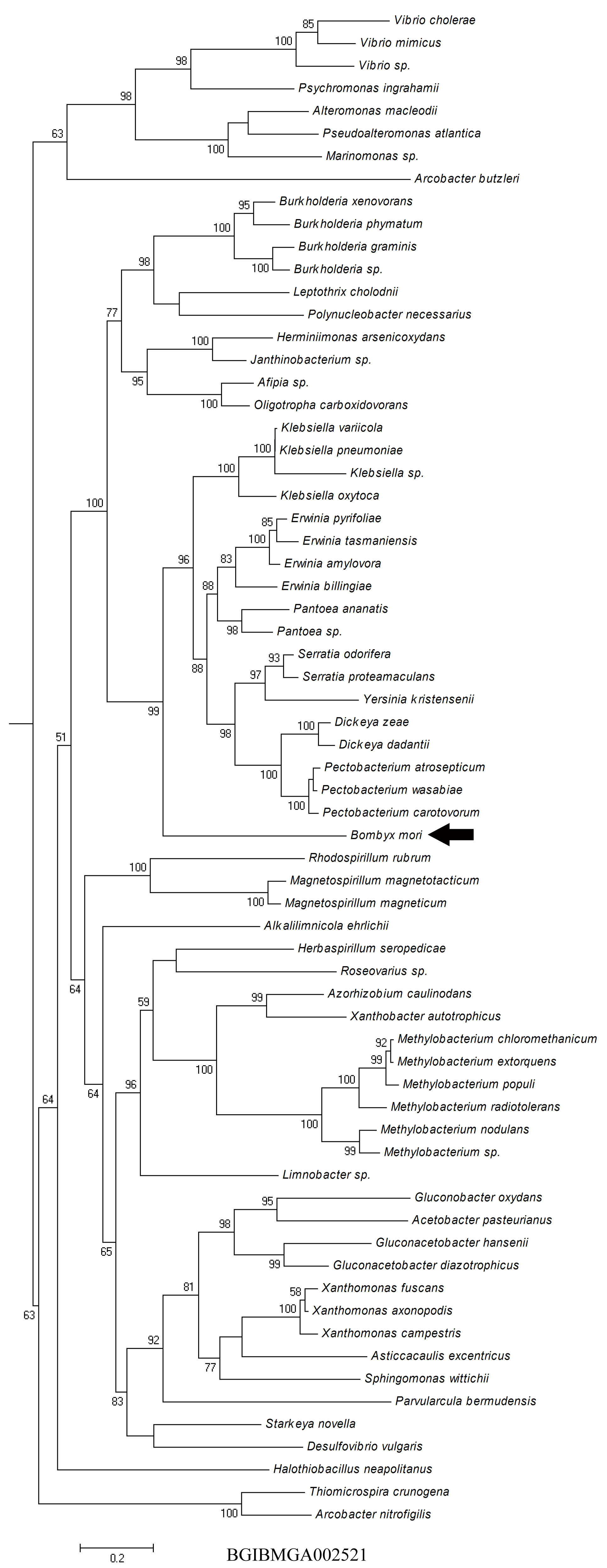

Supplement: Additional file 8 — Phylogenies of other first reported putative transferred genes in Bombyx mori in this paper. [file 1471-2164-12-248-S8.JPEG]

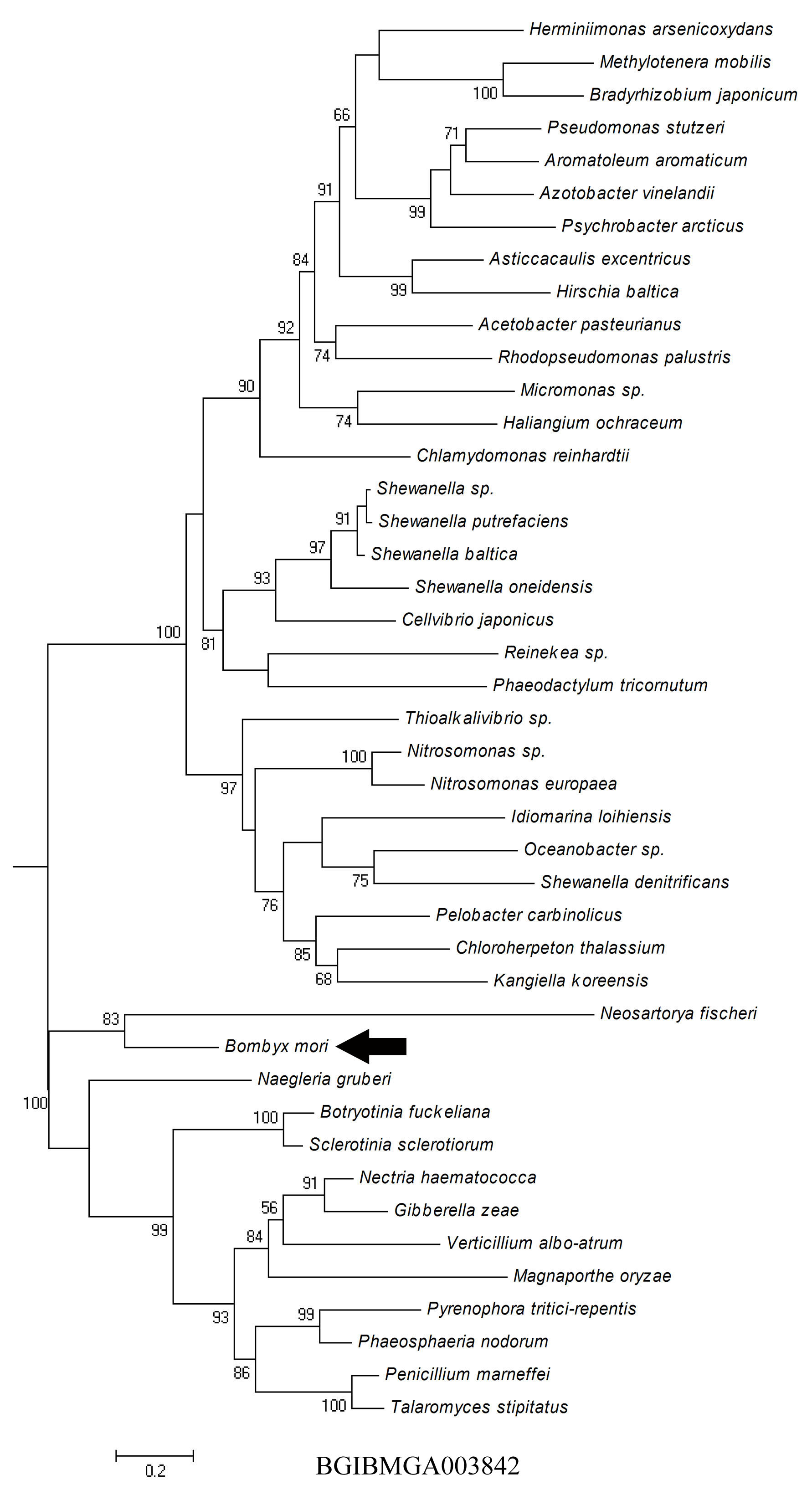

Supplement: Additional file 9 — Phylogenies of other first reported putative transferred genes in Bombyx mori in this paper. [file 1471-2164-12-248-S9.JPEG]
